# Supplementary material for: Rapid, Efficient, and Cost-Effective Gene Editing of Enterococcus faecium with CRISPR-Cas12a
Source: Microbiol Spectr. 2022 Feb 2;10(1):e02427-21. doi: 10.1128/spectrum.02427-21 (PMC8809335; doi:10.1128/spectrum.02427-21)
Supplement: SUPPLEMENTAL FILE 1 — Supplemental material. Download SPECTRUM02427-21_Supp_1_seq2.pdf, PDF file, 0.5 MB [file spectrum02427-21_supp_1_seq2.pdf]

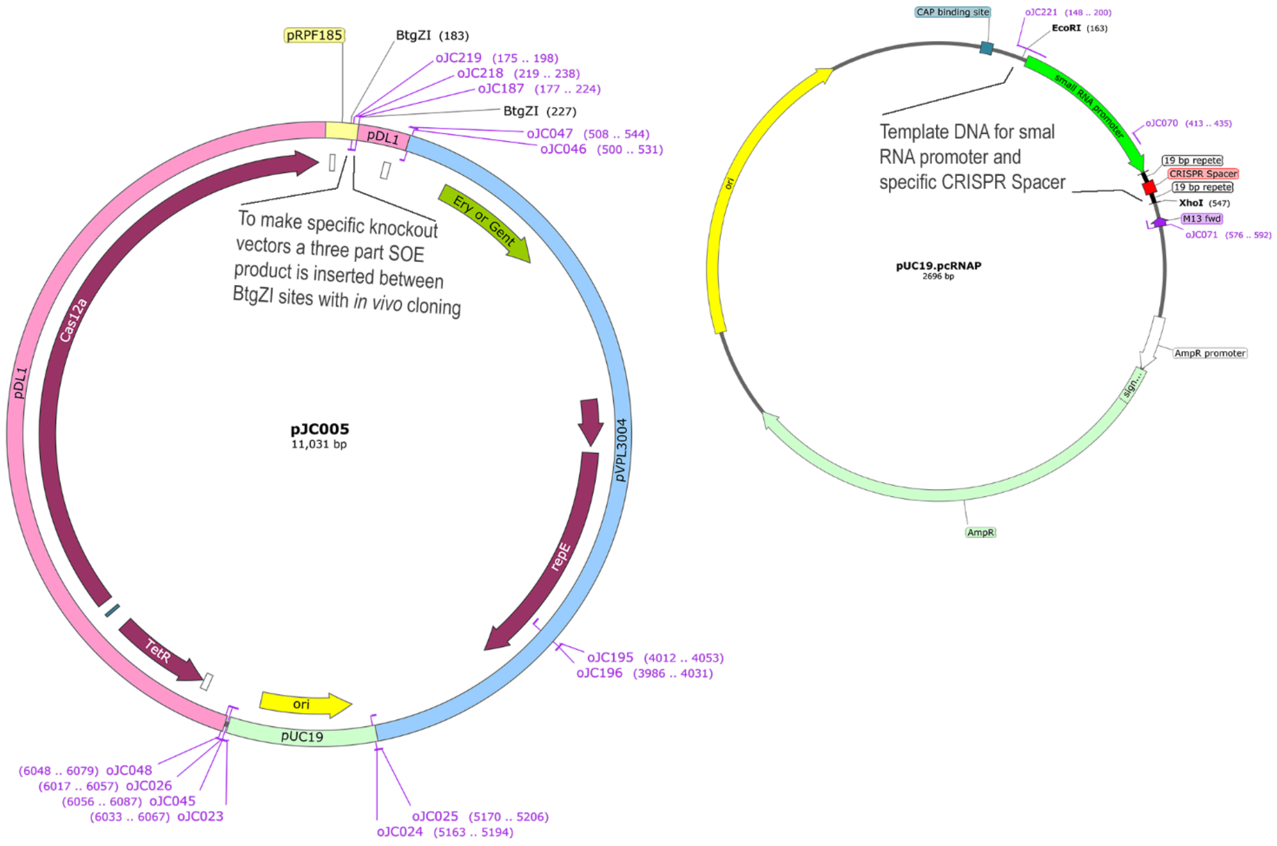

Figure S1 Map of pJC005 & pUC19.pcRNA. The specific SOE PCR product to generate a gene specific knockout/in is inserted following linearization of pJC005 via BtgZI restriction digestion or PCR with oligos oJC218-oJC219. pUC19.pcRNA acts as template DNA for the small RNA promoter and CRISPR Spacer. The CRISPR Spacer is unique to each knockout construct and added to the reverse oligo.

Construction of Plasmids

PCR products were amplified using the Q5 DNA polymerase according to the manufacturer's protocol. Plasmid and genomic DNA concentrations for PCR ranged from 1-10 ng and 150-400 ng, respectively. Primers used to amplify the inserts contained overhangs to allow for *in vivo* assembly. PCR products from plasmid DNA were DpnI-digested to remove template DNA and minimize transformation background. The Zymo DNA Clean & Concentrator™-5 was used to clean all PCR products before use.

To knock out specific genes, pJC005 requires three inserts: a small RNA promoter driving the CRISPR protospacer RNA and up and downstream arms homologous to the flanking regions of the target gene. To act as template DNA, we synthesized a 397 bp small RNA promoter (from *Clostridium beijerinckii*) and nonsense spacer sequence between two 19 bp repeats and cloned the sequence into pUC19 following EcoRI-XhoI digestion generating pUC19.pcRNA.

```
GCTGGAATTCGTCATAATCTTTAATTTGAAAAGATTTAAGGCTTATTTAAATAAAAAATATGAGGGAAGAATTGATA
TAAATTTAATTTTGTTATTGTATTATGGTATGTATGGAATAAATTTAACATAAAGACAGTAATAATGTTCTTGAATT
TAGACTTTTTATGTGTTATCATTAACAAGTATCAAAAATGACATTTAATAAATTAATAATAATTTTAAAAATATATT
TTTGATAAAAGCAATGATTAACATGGTTTGACGTCTGAGAAGAGACGATTTTCTCAATAGGAGAAATTAAGGTGCAA
acccttatcattccaccaTAATTTCTACTCTTGTAGATtcctcaaagagcatatggatatgAATTTCTACTCTTGTA
GATCTCGAGGCC
```

Restriction sites – small RNA promoter - repeats

The CRISPR protospacer is a 23 bp spacer sequence homologous to the target sequence with a protospacer adjacent motif (PAM) of 5`-TTTV-3` immediately upstream. The upstream and downstream sequences are used for homologous recombination and are between 500 and 800 bp.

To knockout the *treA* gene in *E. faecium* NCTC7171, pJC005.X*treA* was generated. Primers oMC046/047 and oMC048/49 were used to amplify the upstream and downstream arms of pJC005.X*treA*, respectively, from *E. faecium* NCTC7171 genomic DNA. Primer oJC221 is a universal primer that binds to the small RNA promoter template DNA and has overhangs homologous to linearized pJC005. Primer oMC045 binds to the small RNA promoter and repeat region, and its overhangs contain the specific *treA* target sequence.

To knockout the *lacL* gene, pJC005.X*lacL* was generated. Primers oMC056/057 and oMC058/59 were used to amplify the upstream and downstream arms, respectively. The small RNA promoter was amplified from pUC19.pcRNA using primers oJC221 and oMC055.

To knockout the *acpH* gene, pJC005.X*acpH* was generated. Primers oJC075/076 and oJC077/78 were used to amplify the upstream and downstream arms, respectively. The small RNA promoter was amplified from pUC19.pcRNA using primers oJC221 and oJC074.

SOE PCR was used to ligate the small RNA promoter containing the CRISPR spacer and the up-and-downstream homologous arms. Individual PCR products were cleaned by column purification and 50 ng of the largest fragment and equimolar amounts of the smaller pieces were mixed in a 20 µl final volume Q5 PCR reaction. The NEB Tm calculator was used to determine annealing temperatures for the following cycling conditions: 98°C for 10 sec, then 10 cycles of 98°C for 10 sec, Tm of homologous regions for 30 sec, 72°C for 30 sec, and a final extension at 72°C for 10 min. Following the first round of SOE PCR, 1 µl of each universal SOE primer (oMC087-oMC088) was added, and the reaction returned to the thermocycler for 98°C for 2 min, then 15 cycles of 98°C for 10 sec, 65°C for 30 sec, 72°C for 30 sec; and a

final extension at 72°C for 10 min. One µl of the SOE PCR product was used directly for *in vivo* cloning if a single band was observed following gel electrophoresis. If multiple bands were present, the correct size band was gel extracted before use.

The knockout plasmids for this study were assembled *in vivo* by High Efficiency NEB® 10-beta Competent *E. coli*. Prior to transformation, 2 µl the SOE PCR product and 50 ng of linearized pJC005 backbone were mixed and added to 50 µl of chemically competent *E. coli* DB10 cells. Cells were incubated on ice for 30 minutes, heat shocked at 42°C for 30 seconds and incubated on ice for another 3 minutes. After addition of 450 µl SOC, the cells were incubated at 37°C with shaking for 1 hour. Transformed cells were selected for on LB agar supplemented with 200 µg/ml erythromycin. Transformants with the correct plasmid were confirmed with primers oJC070 and oJC071.

To knock-in the *unaG* gene, pJC005.X*treA*::*unaG* was generated to replace the *treA* gene with the *unaG* gene. pJC005.X*treA* was linearized in between the *treA* upstream and downstream arms using primers oMC099 and oMC100, both of which contain overhangs for the *unaG* gene. The codon optimized *unaG* gene was amplified using primers oMC101 and oMC102 from pMC001 (*unaG* gene under TetR control, this study). Primers oMC101 and oMC102 have overhangs homologous to the *treA* upstream and downstream arms, respectively. pJC005.*treA*::*unaG* was also assembled *in vivo* by High Efficiency NEB® 10-beta Competent *E. coli*. The transformation protocol resembled the knockout plasmid protocol, and the transformants with the correct plasmid were confirmed with primers oJC070 and oJC071.

Primers used. CRISPR Spacer targets are underlined.

Primer name	Purpose	Primer sequence 5'-3'
oMC046	pJC005.XtreA US arm	GGAGGAATTCGAACAGAATTTCTACTCTTGTAGATCAGGACTGCTGGCTTTTATCG
oMC047		GTTTTGTGTTTTTGTTTTAGGATATTTACCTCCATTCAACTGA
oMC048	pJC005.XtreA DS arm	TTGAATGGAGGTAAATATCCTAAAACAAAAACACAAAACGTCGTTCCT
oMC049		GCATGTCTGCAGGCCTCGAGTTGGGAAAGTGGCGCACAAC
oJC221	Small RNA promoter	GATGCCAGTGTGCTGGAATTCGTCATAATCTTTAATTTGAAAAGATTTAAGGC
oMC045	*treA* CRISPR target	ATTACTGTTCGAATTCCTCCATCGTACATCTACAAGAGTAGAAATTATGGTGGAATGATAAGGGTT
oMC055	*lacL* CRISPR target	ATTACTACTTCTTCCCTTGTTGCATAGATCTACAAGAGTAGAAATTATGGTGGAATGATAAGGGTT
oMC056	pJC005.XlacL US arm	ACAAGGGAAGAAGTAGAATTTCTACTCTTGTAGATCTGTTCTGGTGGAGGTGGAC
oMC057		CGTATCCATATGACTTTTAACTTTTTTCCTTCTCTCTCATAACTTT
oMC058	pJC005.XlacL DS arm	TGAGAGAGAAGGAAAAAAGTTAAAAGTCATATGGATACGGAAAAAGTTGC
oMC059		GCATGTCTGCAGGCCTCGAGCTTCGACACCACCAGCCATC
oJC070	Plasmid confirmation	TGGTTTGACGTCTGAGAAGAGAC
oJC071		GTAAAACGACGGCCAGT
oMC099	pJC005.treA::unaG linearization	TGGAGGTAAATATCCATGGTTGAAAAATTTGTAGG
oMC100		TGTGTTTTTGTTTTATTATTCTGTTGCTCGACGAT
oMC101	Codon-optimized *unaG*	TGGGCAACAGACCTCTTAGAA
oMC102		CCGTAAATTCAAACAACGCTAC
oJC074	*acpH* CRISPR target	ATTAGTTAGGGGATGACCTTTGACTACATCTACAAGAGTAGAAATTATGGTGGAATGATAAGGGTT
oJC075	pJC005.em.XacpH US arm	AAGGTCATCCCCTAACAATTTCTACTCTTGTAGATGTGTCGGCATATGGATCCTCC
oJC076		TATGCATTTATCTCAATTTATGCTTACCTTCTCCTTTTATTGCT
oJC077	pJC005.em.XacpH DS arm	TAAAAGGAGAAGGTAAGCATAAATTGAGATAAATGCATACAAAAGAGACA
oJC078		AAAACGACGGCCAGTGCCAAAAGTTTTGTCGGCGCGGTAG

Sequence of pJC005.em

```
>pJC005(11,031 bp)
TATGACCATGATTACGAATTCGAGCTCGGTACCCGGGGATCCTATAAGTTTTAATAAAACTTTAAATAGAAAAAGGCTTC
TCTCATGAGAAGTCTTTTTTATTTAAAATAAATATAAAATAAAATAGAGGCTATAAATAGCCTCTATTTTATGTGAGAAA
TCCCTAAATAAAAAGATGCCAGTGTGCTGGAATTCGTCATCGCATGAGTCAGCGATGACTCGAGGCCTGCAGACATGCAA
GCTTGGCACTGGCCGTCGTTTTACAACGTCGTGACTGGGAAAACCCTGGCGTTACCCAACTTAATCGCCTTGCAGCACAT
CCCCCTTTCGCCAGCTGGCGTAATAGCGAAGAGGCCCGCACCGATCGCCCTTCCCAACAGTTGCGCAGCCTGAATGGCGA
ATGGCGCTAGCATAAAAATAAGAAGCCTGCATTTGCAGGCTTCTTATTTTTATGGCGCGCCGTTCTGAATCCTTAGCTAA
TGGTTCAACAGGTAACTATGACGAAGATAGCACCCTGGAGCAACCAGGAATGAATTACTATCCCTTTTATCAAGAAGCGC
ACAAAAAGAAAAACGAAATGATACACCAATCAGTGCAAAAAAAGATATAATGGGAGATAAGACGGTTCGTGTTCGTGCTG
ACTTGCACCATATCATAAAAATCGAAACAGCAAAGAATGGCGGAAACGTAAAAGAAGTTATGGAAATAAGACTTAGAAGC
AAACTTAAGAGTGTGTTGATAGTGCAGTATCTTAAAATTTTGTATAATAGGAATTGAAGTTAAATTAGATGCTAAAAATT
TGTAATTAAGAAGGAGTGATTACATGAACAAAAATATAAAATATTCTCAAAACTTTTTAACGAGTGAAAAGTACTCAAC
CAAATAATAAAACAATTGAATTTAAAAGAAACCGATACCGTTTACGAAATTGGAACAGGTAAAGGGCATTTAACGACGAA
ACTGGCTAAAATAAGTAAACAGGTAACGTCTATTGAATTAGACAGTCATCTATTCAACTTATCGTCAGAAAAATTAAAAC
TGAATACTCGTGTCACTTTAATTCACCAAGATATTCTACAGTTTCAATTCCCTAACAAACAGAGGTATAAAATTGTTGGG
AGTATTCCTTACCATTTAAGCACACAAATTATTAAAAAAGTGGTTTTTGAAAGCCATGCGTCTGACATCTATCTGATTGT
TGAAGAAGGATTCTACAAGCGTACCTTGGATATTCACCGAACACTAGGGTTGCTCTTGCACACTCAAGTCTCGATTCAGC
AATTGCTTAAGCTGCCAGCGGAATGCTTTCATCCTAAACCAAAAGTAAACAGTGTCTTAATAAAACTTACCCGCCATACC
ACAGATGTTCCAGATAAATATTGGAAGCTATATACGTACTTTGTTTCAAAATGGGTCAATCGAGAATATCGTCAACTGTT
TACTAAAAATCAGTTTCATCAAGCAATGAAACACGCCAAAGTAAACAATTTAAGTACCGTTACTTATGAGCAAGTATTGT
CTATTTTTAATAGTTATCTATTATTTAACGGGAGGAAATAATTCTATGAGTCGCTTTTGTAAATTTGGAAAGTTACACGT
TACTAAAGGGAATGTAGATAAATTATTAGGTATACTACTGACAGCTTCCAAGGAGCTAAAGAGGTCCCTAGCGCTTAGAA
TCGCTTTAGGAAACACGATCCAGTCCAATAATCGTCGATAAAAACTTTTGAAAAAGGTTGGTGAAATTACCTACTTTTGG
AATAATCACAAATCACAAGTGATTAATCACAAATCACAAGTGATTAATCACTTGTTTATTAAGATATTAAAAGCTATAAT
TTAAATAAAGCGTGAATTTTATTACACAAAAAGAGGGGGGAGAAACTTGGAACTAGCATTTAGAGAAAGCTTAAAAAAGA
TGAGAGGTACCAAATCAAAAGAAAAATTCTCCCAAGAATTAGAAATGAGTAGATCAAATTATTCACGAATAGAATCAGGA
AAATCAGATCCAACCATAAAAACACTAGAACAAATTGCAAAGTTAACTAACTCAACGCTAGTAGTGGATTTAATCCCAAA
TGAGCCAACAGAACCAGAACCAGAAACAGAATCAGAACAAGTAACATTGGATTTAGAAATGGAAGAAGAAAAAAGCAATG
```

ACTTCGTGTGAATAATGCGCGAAATCGTTGCTTATTTTTTTTAAAAGCGGTATACTAGATATAACGAAACAACGAACTGA
ATAGAAACGAAAAAAGAGCCATGACACATTTATAAAATGTTTGACGACATTTTATAAATGCATAGCCCGATAAGATTGCC
AAACCAACGCTTATCAGTTAGTCAGATGAACTCTTCCCTCGTAAGAAGTTATTTAATTAACTTTGTTTGAAGACGGTATA
TAACCGTACTATCATTATATAGGGAAATCAGAGAGTTTTCAAGTATCTAAGCTACTGAATTTAAGAATTGTTAAGCAATC
AATCGGAAATCGTTTGATTGCTTTTTTTGTATTCATTTATAGAAGGTGGAGTTTGTATGAATCATGATGAATGTAAAACT
TATATAAAAAATAGTTTATTGGAGATAAGAAAATTAGCAAATATCTATACACTAGAAACGTTTAAGAAAGAGTTAGAAAA
GAGAAATATCTACTTAGAAACAAAATCAGATAAGTATTTTTCTTCGGAGGGGGAAGATTATATATATAAGTTAATAGAAA
ATAACAAAATAATTTATTCGATTAGTGGAAAAAAATTGACTTATAAAGGAAAAAAATCTTTTTCAAAACATGCAATATTG
AAACAGTTGAATGAAAAAGCAAACCAAGTTAATTAAACAACCTATTTTATAGGATTTATAGGAAAGGAGAACAGCTGAAT
GAATATCCCTTTTGTTGTAGAAACTGTGCTTCATGACGGCTTGTTAAAGTACAAATTTAAAAATAGTAAAATTCGCTCAA
TCACTACCAAGCCAGGTAAAAGCAAAGGGGCTATTTTTGCGTATCGCTCAAAATCAAGCATGATTGGCGGTCGTGGTGTT
GTTCTGACTTCCGAGGAAGCGATTCAAGAAAATCAAGATACATTTACACATTGGACACCCAACGTTTATCGTTATGGAAC
GTATGCAGACGAAAACCGTTCATACACGAAAGGACATTCTGAAAACAATTTAAGACAAATCAATACCTTCTTTATTGATT
TTGATATTCACACGGCAAAAGAAACTATTTCAGCAAGCGATATTTTAACAACCGCTATTGATTTAGGTTTTATGCCTACT
ATGATTATCAAATCTGATAAAGGTTATCAAGCATATTTTGTTTTAGAAACGCCAGTCTATGTGACTTCAAAATCAGAATT
TAAATCTGTCAAAGCAGCCAAAATAATTTCGCAAAATATCCGAGAATATTTTGGAAAGTCTTTGCCAGTTGATCTAACGT
GTAATCATTTTGGTATTGCTCGCATACCAAGAACGGACAATGTAGAATTTTTTGATCCTAATTACCGTTATTCTTTCAAA
GAATGGCAAGATTGGTCTTTCAAACAAACAGATAATAAGGGCTTTACTCGTTCAAGTCTAACGGTTTTAAGCGGTACAGA
AGGCAAAAAACAAGTAGATGAACCCTGGTTTAATCTCTTATTGCACGAAACGAAATTTTCAGGAGAAAAGGGTTTAATAG
GGCGTAATAACGTCATGTTTACCCTCTCTTTAGCCTACTTTAGTTCAGGCTATTCAATCGAAACGTGCGAATATAATATG
TTTGAGTTTAATAATCGATTAGATCAACCCTTAGAAGAAAAAGAAGTAATCAAAATTGTTAGAAGTGCCTATTCAGAAAA
CTATCAAGGGGCTAATAGGGAATACATTACCATTCTTTGCAAAGCTTGGGTATCAAGTGATTTAACCAGTAAAGATTTAT
TTGTCCGTCAAGGGTGGTTTAAATTCAAGAAAAAAAGAAGCGAACGTCAACGTGTTCATTTGTCAGAATGGAAAGAAGAT
TTAATGGCTTATATTAGCGAAAAATCAGATGTATACAAGCCTTATTTAGTGACGACCAAAAAAGAGATTAGAGAAGTGCT
AGGCATTCCTGAACGGACATTAGATAAATTGCTGAAGGTACTGAAGGCGAATCAGGAAATTTTCTTTAAGATTAAACCAG
GAAGAAATGGTGGCATTCAACTTGCTAGTGTTAAATCATTGTTGCTATCGATCATTAAAGTAAAAAAAGAAGAAAAAGAA
AGCTATATAAAGGCGCTGACAAATTCTTTTGACTTAGAGCATACATTCATTCAAGAGACTTTAAACAAGCTAGCAGAACG
CCCTAAAACGGACACACAACTCGATTTGTTTAGCTATGATACAGGCTGAAAATAAAACCCGCACTATGCCATTACATTTA
TATCTATGATACGTGTTTGTTTTTTCTTTGCTGTTTAGCGAATGATTAGCAGAAATATACAGAGTAAGATTTTAATTAAT
TATTAGGGGGAGAAGGAGAGAGTAGCCCGAAAACTTTTAGTTGGCTTGGACTGAACGAAGTGAGGGAAAGGCTACTAAAA
CGTCGAGGGGCAGTGAGAGCGAAGCGAACACTTGATTTTTTAATTTTCTATCTTTTATAGGTCATTAGAGTATACTTATT
TGTCCTATAAACTATTTAGCAGCATAATAGATTTATTGAATAGGTCATTTAAGTTGAGCATATTAGAGGAGGAAAATCTT
GGAGAAATATTTGAAGAACCCGATTACATGGATTGGATTAGTTCTTGTGGTTACGTGGTTTTTAACTAAAAGTAGTGAAT
TTTTGATTTTTGGTGTGTGTGTCTTGTTGTCAGTATTTGCTAGTCAAAGTGATTAAATAGAATTCTCATGTTTGACAGCT
TATCATCGGAGCTCCGATGATAAGCTGTCAAACATGAGAATTCCCGGGGATCCTTACGAAATCATCCTGTGGAGCTTAGT
AGGTTTAGCAAGATGGCAGCGCCTAAATGTAGAATGATAAAAGGATTAAGAGATTAATTTCCCTAAAAATGATAAAACAA
GCGTTTTGAAAGCGCTTGTTTTTTTGGTTTGCAGTCAGAGTAGAATAGAAGTATCAAAAAAAGCACCGACTCGGTGCCAC
TTTTTCAAGTTGATAACGGACTAGCCTTATTTTAACTTGCTATGCTGTTAAGAATGGTTCCAACGCTCACTCAAAGGCGG
TAATACGGTTATCCACAGAATCAGGGGATAACGCAGGAAAGAACATGTGAGCAAAAGGCCAGCAAAAGGCCAGGAACCGT
AAAAAGGCCGCGTTGCTGGCGTTTTTCCATAGGCTCCGCCCCCCTGACGAGCATCACAAAAATCGACGCTCAAGTCAGAG
GTGGCGAAACCCGACAGGACTATAAAGATACCAGGCGTTTCCCCCTGGAAGCTCCCTCGTGCGCTCTCCTGTTCCGACCC
TGCCGCTTACCGGATACCTGTCCGCCTTTCTCCCTTCGGGAAGCGTGGCGCTTTCTCATAGCTCACGCTGTAGGTATCTC
AGTTCGGTGTAGGTCGTTCGCTCCAAGCTGGGCTGTGTGCACGAACCCCCCGTTCAGCCCGACCGCTGCGCCTTATCCGG
TAACTATCGTCTTGAGTCCAACCCGGTAAGACACGACTTATCGCCACTGGCAGCAGCCACTGGTAACAGGATTAGCAGAG
CGAGGTATGTAGGCGGTGCTACAGAGTTCTTGAAGTGGTGGCCTAACTACGGCTACACTAGAAGAACAGTATTTGGTATC
TGCGCTCTGCTGAAGCCAGTTACCTTCGGAAAAAGAGTTGGTAGCTCTTGATCCGGCAAACAAACCACCGCTGGTAGCGG
TGGTTTTTTTGTTTGCAAGCAGCAGATTACGCGCAGAAAAAAAGGATCTCAAGAAGATCCTTTGATCTTTTCTACGGGGT
CTGACGCTCAGTGGAACGAAAACTCACGTTAAGGGATTTTGGTCATGAGATTATCAAAAAGGATCTTCACCTAGATCCTT
TTAAATTAAAAATGAAGTTTTAAATCAATCTAAAGTATATATGAGTAAAGATGTGATCCGTAGCGGTATGCGCTCCATCA
AGAAGAGCGACTTCGCGGAGCTGGTGAAGTACATCACCGACGAGCAAGGCAAGACCGATCGGGCCCCCTGCAGGATAAAA
AAATTGTAGATAAATTTTATAAAATAGTTTTATCTACAATTTTTTTATCAGGAAACAGCTATGACCGCGGCCGCTGTATC
CACATAAAAATAAGAAGCCTGCATTTGCAGGCTTCTTATTTTTATGGTACCTTAAGACCCACTTTCACATTTAAGTTGTT
TTTCTAATCCGCATATGATCAATTCAAGGCCGAATAAGAAGGCTGGCTCTGCACCTTGGTGATCAAATAATTCGATAGCT
TGTCGTAATAATGGCGGCATACTATCAGTAGTAGGTGTTTCCCTTTCTTCTTTAGCGACTTGATGCTCTTGATCTTCCAA
TACGCAACCTAAAGTAAAATGCCCCACAGCGCTGAGTGCATATAATGCATTCTCTAGTGAAAAACCTTGTTGGCATAAAA
AGGCTAATTGATTTTCGAGAGTTTCATACTGTTTTTCTGTAGGCCGTGTACCTAAATGTACTTTTGCTCCATCTCGGTGA
CTTAGTAAAGCACATCTAAAACTTTTAGCGTTATTACGTAAAAAATCTTGCCAGCTTTCCCCTTCTAAAGGGCAAAAGTG
AGTATGGTGCCTATCTAACATCTCAATGGCTAAGGCGTCGAGCAAAGCCCGCTTATTTTTTACATGCCAATACAATGTAG
GCTGCTCTACACCTAGCTTCTGGGCGAGTTTACGGGTTGTTAAACCTTCGATTCCGACCTCATTAAGCAGCTCTAATGCG
CTGTTAATCACTTTACTTTTATCTAATCTAGACATCATTAATTCCTCCTTTTTGTTGACATTATATCATTGATAGAGTTA
TTTGTCAAACTAGTTTTTTATTTCGATGCCCTGGACTTCATGAAAAACTAAAAAAAAATATTGACACTCTATCATTGATAG
AGTATAATTAAAATAAGCTTGATCGTAGCGTTAACAGATCTGAGCTCCTGCAGTAAAGGAGGTTTTTATGACACAGTTTG
AAGGCTTCACCAATCTCTACCAGGTCAGCAAGACGCTACGTTTTGAGCTTATCCCGCAGGGAAAAACCCTGAAACACATT

CAGGAACAGGGGTTCATAGAGGAAGATAAGGCGCGTAACGACCATTATAAAGAACTGAAGCCTATAATCGACCGTATTTA
TAAAACGTACGCGGATCAGTGCCTGCAGCTGGTTCAGCTGGATTGGGAGAATCTGTCCGCGGCTATTGATAGCTATCGCA
AAGAGAAGACCGAGGAAACCCGTAACGCACTGATTGAAGAGCAGGCGACCTATCGGAATGCGATCCATGATTACTTCATC
GGCCGCACCGACAACCTGACCGATGCAATTAACAAACGTCACGCAGAGATTTACAAAGGTCTGTTTAAAGCAGAGTTATT
CAATGGCAAGGTTCTGAAACAGCTGGGTACGGTCACCACCACCGAACACGAAAACGCACTGCTGAGGAGCTTTGATAAAT
TTACCACATATTTCAGCGGTTTCTATGAAAATCGTAAGAATGTATTTAGCGCCGAAGATATTTCCACCGCAATTCCTCAT
CGTATTGTGCAGGATAATTTTCCGAAGTTTAAAGAAAATTGTCATATTTTTACCCGTCTGATCACCGCGGTACCGAGCCT
GCGAGAGCATTTTGAAAACGTTAAGAAAGCCATTGGAATTTTTGTCAGTACCAGCATTGAAGAAGTGTTTTCGTTCCCGT
TCTATAACCAACTGCTGACCCAGACCCAGATTGATCTGTACAATCAGCTGCTGGGGGGCATAAGCCGCGAGGCAGGTACC
GAAAAGATAAAGGGACTCAATGAGGTGCTGAATCTGGCAATTCAGAAGAATGATGAAACGGCTCATATCATTGCTAGCCT
GCCGCATCGTTTCATTCCCCTGTTTAAGCAAATCCTGAGCGATCGCAATACACTGAGCTTTATCCTCGAAGAGTTTAAAT
CGGACGAAGAAGTTATCCAGAGCTTTTGCAAATACAAAACCCTGCTGCGGAACGAAAATGTGCTGGAGACCGCTGAAGCA
CTGTTTAATGAACTGAACTCGATCGACCTCACCCATATTTTTATATCCCACAAAAAACTGGAAACCATAAGCAGCGCTCT
GTGTGACCATTGGGATACCCTGCGCAACGCCCTGTATGAACGGCGTATCAGCGAGCTGACCGGGAAAATCACCAAATCCG
CAAAGGAAAAAGTTCAGCGTAGTCTGAAACACGAGGACATCAACCTGCAAGAAATTATTAGCGCAGCAGGTAAAGAGCTG
AGCGAAGCATTCAAACAGAAAACCAGCGAAATCCTGAGCCATGCCCATGCTGCACTGGATCAGCCGCTGCCGACCACCCT
GAAAAAACAGGAGGAAAAGGAGATTCTGAAAAGCCAACTGGACAGCCTGCTGGGCCTGTATCACCTGCTGGACTGGTTTG
CAGTCGATGAGAGCAACGAGGTTGATCCTGAGTTCTCCGCTCGTCTGACCGGAATCAAGCTGGAGATGGAACCGAGTCTG
TCGTTTTACAATAAAGCGCGTAATTACGCGACCAAGAAACCGTATAGCGTGGAAAAATTCAAACTGAACTTTCAGATGCC
GACCCTTGCAAGCGGATGGGACGTTAACAAAGAAAAAAACAATGGGGCAATTCTGTTTGTGAAAAATGGCCTCTATTATC
TGGGTATCATGCCGAAACAGAAAGGGCGCTACAAAGCCCTGTCATTTGAGCCGACCGAGAAAACCTCAGAGGGTTTCGAC
AAGATGTACTACGATTATTTCCCGGATGCGGCAAAAATGATACCCAAATGTAGCACCCAACTGAAGGCAGTTACAGCCCA
CTTTCAGACCCATACCACCCCGATCCTGCTGTCGAACAATTTTATAGAGCCGCTGGAAATTACCAAAGAGATTTATGATC
TGAATAATCCGGAAAAGGAGCCCAAGAAATTTCAGACGGCGTATGCAAAAAAGACCGGGGATCAGAAAGGTTATCGTGAA
GCGCTGTGCAAATGGATTGACTTTACCCGTGACTTTCTGTCAAAATATACCAAAACGACGAGCATTGATCTGAGCAGCCT
ACGTCCGAGCAGCCAATATAAGGATCTGGGCGAATATTACGCCGAACTGAATCCGCTGCTCTACCATATTTCCTTCCAAC
GAATCGCTGAAAAAGAAATAATGGACGCCGTTGAAACCGGCAAACTGTATCTGTTTCAAATCTACAACAAAGATTTCGCC
AAAGGCCATCACGGTAAGCCGAACCTGCATACCCTGTATTGGACCGGTCTGTTTAGCCCGGAGAATCTGGCCAAAACCAG
CATCAAGCTGAACGGACAGGCAGAACTGTTTTACCGCCCCAAAAGCCGTATGAAAAGGATGGCACACCGCCTGGGCGAAA
AAATGCTGAATAAGAAACTCAAAGATCAGAAAACGCCGATACCGGATACCCTTTATCAGGAGCTGTATGATTATGTTAAC
CACCGGCTGAGCCATGACCTGAGCGACGAAGCGCGTGCACTGCTGCCGAACGTGATTACCAAGGAAGTCTCGCATGAAAT
TATTAAAGATCGGCGCTTCACCAGTGATAAATTTTTCTTCCATGTACCGATCACCCTGAATTATCAAGCCGCAAATAGCC
CTTCCAAATTTAATCAACGCGTGAATGCGTACCTGAAAGAGCATCCGGAGACCCCAATTATTGGCATAGACCGAGGAGAA
CGCAATCTCATTTATATCACCGTCATTGATAGCACCGGTAAGATCCTGGAACAGCGTAGCCTGAATACCATTCAGCAGTT
TGACTACCAGAAAAAGCTGGACAACAGAGAAAAGGAACGTGTAGCCGCCCGGCAGGCTTGGAGTGTGGTGGGTACTATCA
AGGATCTGAAGCAGGGGTATCTCTCCCAAGTTATCCATGAAATTGTCGATCTAATGATTCACTATCAAGCAGTAGTGGTA
CTGGAAAATCTGAATTTCGGTTTCAAAAGCAAACGTACAGGGATCGCTGAAAAAGCCGTTTATCAGCAGTTCGAGAAAAT
GCTGATAGACAAGCTGAATTGCCTGGTTCTGAAAGATTATCCGGCAGAGAAGGTGGGCGGTGTGCTGAACCCGTACCAGC
TGACTGATCAATTTACGAGCTTTGCAAAAATGGGAACGCAGAGCGGTTTCCTGTTCTATGTTCCGGCGCCATATACCAGC
AAGATAGACCCGCTGACAGGTTTCGTAGATCCGTTTGTCTGGAAAACCATTAAAAATCATGAAAGTCGCAAACATTTTCT
GGAGGGCTTTGATTTTCTGCACTATGACGTGAAAACCGGCGACTTCATTCTGCATTTTAAAATGAACCGTAATCTGTCCT
TTCAGCGCGGCCTGCCTGGCTTTATGCCGGCGTGGGACATTGTTTTTGAAAAGAATGAGACACAGTTTGATGCCAAAGGT
ACCCCCTTTATTGCGGGGAAACGCATTGTGCCCGTTATAGAAAATCACCGCTTCACCGGACGGTATAGGGACTTGTACCC
GGCAAATGAATTGATAGCGCTGCTGGAGGAGAAAGGTATTGTCTTTCGGGATGGATCAAACATCCTGCCGAAGCTGCTGG
AGAACGATGACAGCCACGCAATAGACACCATGGTAGCGCTGATCCGAAGCGTGCTGCAGATGCGTAACAGTAATGCGGCT
ACGGGGGAAGACTACATTAATAGCCCGGTCCGTGATCTGAACGGCGTTTGTTTCGATAGCAGATTTCAAAATCCGGAGTG
GCCGATGGATGCCGATGCCAATGGAGCTTACCATATCGCTCTCAAAGGTCAGCTCCTACTGAACCATTTGAAAGAATCAA
AAGATCTGAAACTGCAGAACGGCATCTCGAATCAGGACTGGCTGGCCTACATTCAAGAACTGAGAAACTAA

Sequence of pUC19.pcRNA

>pUC19.pcRNAP(2696 bp)
CCCCGCGCGTTGGCCGATTCATTAATGCAGCTGGCACGACAGGTTTCCCGACTGGAAAGCGGGCAGTGAGCGCAACGCAA
TTAATGTGAGTTAGCTCACTCATTAGGCACCCCAGGCTATTTTATGTGAGAAATCCCTAAATAAAAAGATGCCAGTGTGC
TGGAATTCGTCATAATCTTTAATTTGAAAAGATTTAAGGCTTATTTAAATAAAAAATATGAGGGAAGAATTGATATAAAT
TTAATTTTGTTATTGTATTATGGTATGTATGGAATAAATTTAACATAAAGACAGTAATAATGTTCTTGAATTTAGACTTT
TTATGTGTTATCATTAACAAGTATCAAAAATGACATTTAATAAATTAATAATAATTTTAAAAATATATTTTTGATAAAAG
CAATGATTAACATGGTTTGACGTCTGAGAAGAGACGATTTTCTCAATAGGAGAAATTAAGGTGCAAACCCTTATCATTCC
ACCATAATTTCTACTCTTGTAGATTCCTCAAAGAGCATATGGATATGAATTTCTACTCTTGTAGATCTCGAGGCCTGCAG
ACATGCAAGCTTGGCACTGGCCGTCGTTTTACAACGTCGCAGACAAGCTGTGACCGTCTCCGGGAGCTGCATGTGTCAGA
GGTTTTCACCGTCATCACCGAAACGCGCGAGACGAAAGGGCCTCGTGATACGCCTATTTTTATAGGTTAATGTCATGATA
ATAATGGTTTCTTAGACGTCAGGTGGCACTTTTCGGGGAAATGTGCGCGGAACCCCTATTTGTTTATTTTTCTAAATACA
TTCAAATATGTATCCGCTCATGAGACAATAACCCTGATAAATGCTTCAATAATATTGAAAAAGGAAGAGTATGAGTATTC

AACATTTCCGTGTCGCCCTTATTCCCTTTTTTGCGGCATTTTGCCTTCCTGTTTTTGCTCACCCAGAAACGCTGGTGAAA
GTAAAAGATGCTGAAGATCAGTTGGGTGCACGAGTGGGTTACATCGAACTGGATCTCAACAGCGGTAAGATCCTTGAGAG
TTTTCGCCCCGAAGAACGTTTTCCAATGATGAGCACTTTTAAAGTTCTGCTATGTGGCGCGGTATTATCCCGTATTGACG
CCGGGCAAGAGCAACTCGGTCGCCGCATACACTATTCTCAGAATGACTTGGTTGAGTACTCACCAGTCACAGAAAAGCAT
CTTACGGATGGCATGACAGTAAGAGAATTATGCAGTGCTGCCATAACCATGAGTGATAACACTGCGGCCAACTTACTTCT
GACAACGATCGGAGGACCGAAGGAGCTAACCGCTTTTTTGCACAACATGGGGGATCATGTAACTCGCCTTGATCGTTGGG
AACCGGAGCTGAATGAAGCCATACCAAACGACGAGCGTGACACCACGATGCCTGTAGCAATGGCAACAACGTTGCGCAAA
CTATTAACTGGCGAACTACTTACTCTAGCTTCCCGGCAACAATTAATAGACTGGATGGAGGCGGATAAAGTTGCAGGACC
ACTTCTGCGCTCGGCCCTTCCGGCTGGCTGGTTTATTGCTGATAAATCTGGAGCCGGTGAGCGTGGGTCTCGCGGTATCA
TTGCAGCACTGGGGCCAGATGGTAAGCCCTCCCGTATCGTAGTTATCTACACGACGGGGAGTCAGGCAACTATGGATGAA
CGAAATAGACAGATCGCTGAGATAGGTGCCTCACTGATTAAGCATTGGTAACTGTCAGACCAAGTTTACTCATATATACT
TTAGATTGATTTAAAACTTCATTTTTAATTTAAAAGGATCTAGGTGAAGATCCTTTTTGATAATCTCATGACCAAAATCC
CTTAACGTGAGTTTTCGTTCCACTGAGCGTCAGACCCCGTAGAAAAGATCAAAGGATCTTCTTGAGATCCTTTTTTTCTG
CGCGTAATCTGCTGCTTGCAAACAAAAAAACCACCGCTACCAGCGGTGGTTTGTTTGCCGGATCAAGAGCTACCAACTCT
TTTTCCGAAGGTAACTGGCTTCAGCAGAGCGCAGATACCAAATACTGTTCTTCTAGTGTAGCCGTAGTTAGGCCACCACT
TCAAGAACTCTGTAGCACCGCCTACATACCTCGCTCTGCTAATCCTGTTACCAGTGGCTGCTGCCAGTGGCGATAAGTCG
TGTCTTACCGGGTTGGACTCAAGACGATAGTTACCGGATAAGGCGCAGCGGTCGGGCTGAACGGGGGGTTCGTGCACACA
GCCCAGCTTGGAGCGAACGACCTACACCGAACTGAGATACCTACAGCGTGAGCTATGAGAAAGCGCCACGCTTCCCGAAG
GGAGAAAGGCGGACAGGTATCCGGTAAGCGGCAGGGTCGGAACAGGAGAGCGCACGAGGGAGCTTCCAGGGGGAAACGCC
TGGTATCTTTATAGTCCTGTCGGGTTTCGCCACCTCTGACTTGAGCGTCGATTTTTGTGATGCTCGTCAGGGGGGCGGAG
CCTATGGAAAAACGCCAGCAACGCGGCCTTTTTACGGTTCCTGGCCTTTTGCTGGCCTTTTGCTCACATGTTCTTTCCTG
CGTTATCCCCTGATTCTGTGGATAACCGTATTACCGCCTTTGAGTGAGCTGATACCGCTCGCCGCAGCCGAACGACCGAG
CGCAGCGAGTCAGTGAGCGAGGAAGCGGAAGAGCGCCCAATACGCAAACCGCCTCT
